# Moving to action in the Eastern Mediterranean Region

**DOI:** 10.1007/s00038-017-1013-2

**Published:** 2017-08-03

**Authors:** Ala Alwan

**Affiliations:** Regional Director Emeritus, World Health Organization Eastern Mediterranean Region, Cairo, Egypt

This series of papers comes at a time when there are major challenges facing health development in the Eastern Mediterranean region. Without understanding the challenges facing populations in this region, one cannot identify the way forward in improving their health and fulfilling their aspirations.

One of the characteristics of the region, which strikes anyone who lives in it, or who has visited it extensively, is its diversity and heterogeneity, as well as its commonalities. Although many countries have made great gains and have built extensive modern networks of health infrastructure, these gains have not been shared across and within countries. In our work in the region, we opted for grouping the countries into three groups based on several health outcomes and health system performance indicators (WHO Regional Office for the Eastern Mediterranean 2012a).

Despite the heterogeneity of the region in socioeconomic terms and health indicators, health policy makers agreed since 2012 on five key health priorities that are shared, to varying degrees by all countries (WHO Regional Office for the Eastern Mediterranean 2012b). These priorities, which cover the health conditions reviewed in this issue, are health security and communicable diseases, the non-communicable diseases epidemic, the high burden of maternal and child mortality, emergency preparedness and response and the overarching challenge of health system strengthening and universal health coverage.

In moving forward to address these priorities, I wish to make the following points:

*First*: Sustained political commitment is necessary. The consensus on the five groups of priorities is based on clear understanding of the disease burden and its socioeconomic impact on the populations. We know the main impediments to health development and we know most of the solutions. However, progress depends on the level of political commitments, improved governance, and translation of commitments into sustained action. Implementation also depends on other factors, most important of which is leadership and greater public investment in health—and at the heart of such investment is human capital.

*Second*: Capacity. It is true that massive gains have been made in many countries in provision of curative care; these achievements are not always accompanied by similar progress in promoting health and preventing disease. Health systems in most countries suffer from limited capacity in public health, impeding the implementation of programs that address the key health challenges (WHO Regional Office for the Eastern Mediterranean 2013, 2017). It is important that countries put in place programs and incentives to attract and train the brightest and the best of their young people into public health. If we do not do this, improvements in population health will stall, and countries will continue to struggle to meet the targets set globally and for themselves. To kick start such a move, WHO worked with two countries—Qatar and Morocco—to assess the public health functions of their ministries of health, analyzing strengths and gaps and recommending approaches to strengthen capacity for the future (Alwan et al. 2016). I hope all countries will follow these successful examples.

*Third*: Engagement. The challenges facing health development in the region require a multi-sectoral approach. We will not make a significant difference in any of these priorities without meaningful engagement of non-health sectors and adoption of a whole of government approach (WHO Regional Office for the Eastern Mediterranean 2016a). Addressing health issues in isolation from economic, social and environmental development imperatives will not lead to improved population health.

*Fourth*: Unfortunately, the Eastern Mediterranean Region today suffers from the most serious and most destructive emergencies and crises worldwide (World Health Organization 2017) and has the largest number of refugees and displaced population (The United Nations Refugee Agency 2016). We have seen in this region how public health gains, developed after decades of hard work and investment, are wiped away in just a few months (WHO Syria Country Office 2015; WHO Regional Office for the Eastern Mediterranean 2016b). People are increasingly now living in a region in which a state of emergency seems almost to have become a way of life. While building capacity in emergency response is top priority for countries experiencing crises, it is equally essential for all countries to strengthen their national and community systems by conducting an objective and comprehensive risk and vulnerability assessment and taking robust multi-sectoral action to reinforce emergency preparedness.

Finally, solidarity. To achieve real gains across the five strategic priority areas I have outlined, we need solidarity between all countries. A health threat on the border of one district, one country or one region will not remain in that one place without coordination, collaboration and mutual support. Health security is the concern of every nation. Irrespective of the health threats that may be faced, low-resource countries will require intensive and sustained support from other countries in the same region. This region has countries with the highest income but remains one of the regions of the world with the lowest investment in the contribution to sustainable development provided to their less fortunate neighboring states. I hope this pattern will change and there will be greater solidarity between countries for the sake of health security and development of all their people.
